# Plasma levels of alarmin HNPs 1–3 associate with lung dysfunction after cardiac surgery in children

**DOI:** 10.1186/s12890-017-0558-4

**Published:** 2017-12-28

**Authors:** XiWang Liu, QiXing Chen, YuJia Luo, YaoQin Hu, DengMing Lai, XiaoLe Zhang, XiangHong Zhang, JianGen Yu, XiangMing Fang, Qiang Shu

**Affiliations:** 1 Department of Thoracic & Cardiovascular Surgery, Children’s Hospital, Zhejiang University School of Medicine and Zhejiang Key Laboratory for Diagnosis and Therapy of Neonatal Diseases, 3333 Binsheng Road, Hangzhou, 310003 China; 2 Department of Anesthesiology, the First Affiliated Hospital, Zhejiang University School of Medicine, Hangzhou, 310003 China

**Keywords:** Human neutrophil peptides (HNPs) 1–3, Alarmin, Lung injury, Cardiac surgery, Cardiopulmonary bypass, Infant, Young children, Congenital heart disease

## Abstract

**Background:**

Early onset of lung injury is considerable common after cardiac surgery and is associated with increasing in morbidity and mortality, but current clinical predictors for the occurrence of this complication always have limited positive warning value. This study aimed to evaluate whether elevated plasma levels of human neutrophil peptides (HNPs) 1–3 herald impaired lung function in infants and young children after cardiac surgery necessitating cardiopulmonary bypass (CPB).

**Methods:**

Consecutive children younger than 3 years old who underwent cardiac surgery were prospectively enrolled. Plasma concentrations of HNPs 1–3 and inflammatory cytokines were measured before, and immediately after CPB, as well as at 1 h, 12 h, and 24 h after CPB.

**Results:**

Thirty patients were enrolled, 18 (60%) of whom were infants. Plasma levels of HNPs 1–3 and the pro-inflammatory cytokine interleukin-6 (IL-6) significantly increased immediately after CPB (*P* < 0.001), while IL-8 increased 1 h after the CPB operation (*P* = 0.002). The anti-inflammatory cytokine IL-10 levels were also significantly elevated immediately after CPB compared with the baseline (P < 0.001). The stepwise multiple linear regression analysis showed that the plasma HNPs 1–3 levels immediately after CPB was independent correlated with the declined lung function, as reflected by the PaO_2_/FiO_2_ ratio on the first 2 days after operation (for the first day: OR, −1.067, 95% CI, −0.548 to −1.574; P < 0.001; for the second day: OR, −0.667, 95% CI, −0.183 to −1.148; *P* = 0.009) and prolonged mechanical ventilation time (OR, 0.039, 95% CI, 0.005 to 0.056; *P* = 0.011). Plasma levels of HNPs 1–3 and IL-10 returned to the baseline values, while IL-6 and IL-8 levels remained significantly higher than baseline 24 h after CPB (*P* ≤ 0.01).

**Conclusions:**

Elevated HNPs 1–3 levels immediately after CPB correlate with impaired lung function, and HNPs 1–3 could serve as a quantifiable early alarmin biomarker for onset of lung injury in infants and young children undergoing cardiac surgery with CPB.

**Electronic supplementary material:**

The online version of this article (10.1186/s12890-017-0558-4) contains supplementary material, which is available to authorized users.

## Background

Early onset of postoperative lung injury may occur in 12% to 50% of patients undergoing cardiac surgery necessitating cardiopulmonary bypass (CPB), with clinical manifestations ranging from mild postoperative dyspnoea to florid Acute Respiratory Distress Syndrome (ARDS) [1–3]. Up to 20% of the patients need ventilation for more than 48 h [3, 4]. The ARDS, appearance in 2% of these cases, carries a mortality of 40% to 80% [1, 5]. The etiology of post-CPB pulmonary dysfunction is multifactorial. Despite advances in perioperative management strategies [6], younger children are more prone to postoperative lung injury duo to the detrimental stimulation during cardiac surgery with CPB. Lung injury after surgery often leads to a prolonged length of hospital stay and subsequently increases in therapeutic costs and mortality [7–9]. Ideal therapy is limited by an incomplete understanding of the sentinel events involved in declined lung function, including both the relevant soluble mediators and cognate receptors that are operational when the process is most likely to be reversible [1, 10, 11]. The earlier the lung injury be detected, the much more successful treatment will be done [3, 8, 12]. Therefore, discovery and validation of certain alarmin biomarker for CPB-related lung injury in infants and young children undergoing cardiac surgery would be of great help for early diagnosis and efficient therapeutic decision making.

Human α-defensins, a closely related family of six multifunctional cationic peptides, were the first endogenous mediators to be characterized as alarmins [13–15]. α-defensins 1–3, also called human neutrophil peptides (HNPs) 1–3, are sequestered within intracellular granules and released when polymorphonuclear leukocytes (PMNs) are activated [15, 16]. Elevated levels of HNPs 1–3 have been found in the plasma and bronchoalveolar lavage fluid (BALF) of patients with inflammatory lung disease [17–20]. Furthermore, increased HNPs 1–3 levels are significantly correlated with the severity of lung function decline and clinical outcomes after acute lung injury [18, 20–23]. Recent studies have demonstrated that the release of HNPs 1–3 by activated PMNs contributes to the initiation of acute lung injury by increasing both inflammatory response (activated inflammatory cells and immune recruitment, cytokine and chemokine production) and lung (microvascular and epithelial) permeability [24–26], as well as inducing apoptosis of the bronchial alveolar epithelial cells [26]. In addition, HNPs 1–3 could also suppress neutrophil apoptosis via the P2Y_6_ signaling pathway, which could leads to the amplification of the uncontrolled inflammation in lung [27], for neutrophils play a critical role as effector cells during acute lung injury.

These findings suggest that alarmin HNPs 1–3 may play a key role in the intricate of lung inflammatory response and contribute to the pathogenesis of lung injury. Initial studies are promising. However, it is not clear whether these findings could extend to younger children undergoing cardiac surgery with CPB. This pilot study aimed to characterize the temporal kinetics of plasma alarmin HNPs 1–3 in infants and young children undergoing cardiac surgery with CPB, and to investigate whether plasma HNPs 1–3 levels are associated with the impaired lung function after cardiac surgery.

## Methods

### Study population

This prospective study was conducted at a 1900-bed university children’s hospital located in eastern China. The study protocols were approved by the hospital ethics committee (Medical Ethical Committee of the Children’s Hospital of Zhejiang University), and informed consents were signed by supervisors of the patients. Children who were younger than 3 years and scheduled for cardiac surgery for congenital heart disease (CHD) were consecutively enrolled. The included patients had stable clinical conditions for at least 1 month. Patients were excluded if they were premature, had abnormal liver or renal function, had major chromosomal abnormalities, exhibited pulmonary inflammation before the surgery, had pulmonary edema due to cardiac dysfunction, required extracorporeal membrane oxygenation support after the operation, died because of cardiac dysfunction, or refused to participate in the study.

### Data collection and definitions

During the surgical procedure, all patients underwent routine hemodynamic and blood gas surveillance. Anesthesia, the CPB procedure, ventilator settings and weaning from mechanical ventilation (MV) in the cardiac Intensive Care Unit (CICU) were all performed by using standard protocols, as shown in our previous studies and in additional material in detail [Additional file 1 and Additional file 2] [9, 28]. The patients were transferred to the CICU immediately after operation and were given mechanical ventilated, still sedated and intubated.

Demographic and preoperative data were collected, including the patient’s gender, age, weight, pulse oximetry saturation (SpO_2_) at room air, CPB time, aortic cross-clamp (AC) time, operation time and duration of MV. The Risk Adjusted Classification for Congenital Heart Surgery (RACHS-1) for the operation was 2 to 4 degree. Arterial blood gas surveillance was done within 30 min after mechanical ventilated and then every 4 h. The ratio of fraction of inspired oxygen to oxygen pressure (PaO_2_/FiO_2_) was calculated. It would be checked if the PaO_2_/FiO_2_ was not dissatisfaction and also be done at any time according to the situation. The worst value of PaO_2_/FiO_2_ was record for analysis. Echocardiography was performed routinely to evaluate the cardiac function after surgery and at any time if necessary. The left ventricular function was evaluated according to the ejection fraction (EF). A bedside chest radiograph was taken every day after surgery. Patients with cardiogenic pulmonary edema (CPE) were excluded. CPE was identified when the pulmonary arterial occlusion pressure was >18 mmHg or by the presence of at least two of the following: central venous pressure > 14 mmHg left ventricular EF < 45%, systemic hypertension, or volume overload. Volume overload means cumulative fluid intake (including IV-administered medication, colloid, crystalloid and blood products) exceeding cumulative output (including urine, blood loss) during the observing period. Volume was given at the discretion of the attending cardiac intensivist or cardiovascular surgeon to maintain adequate circulatory parameters. In addition, all patients were followed up for CICU length of stay (LOS) and hospital LOS.

### Determination of HNP 1–3 and inflammatory cytokines in plasma

For each patient, 2 ml of fresh blood was drawn into a vacuum tube containing EDTA at the following time points: before CPB, immediately after CPB, as well as 1 h, 12 h, and 24 h after CPB respectively. After being centrifuged at 3000 rpm for 15 min at 4 °C, the plasma was divided into aliquots and frozen at −80 °C until assay.

HNP 1–3 levels were measured by using the commercially available ELISA kits (ALPHA DIAGNOSTIC INTERNATIONAL, USA), according to the manufacturer’s instructions. The inflammation cytokines plasma levels were also determined with the ELISA kits (R&D Systems, USA), which include IL-6, IL-8, IL-10, IL-1β and TNF-α. All the kits standards and samples run in duplicate. Laboratory staffs were blinded to clinical paraments of the patients, and investigators involved in collecting the demographic and preoperative data were blinded to cytokines levels.

### Statistical analysis

Continuous data were tested for normal distribution with the one-sample Kolmogorov-Smirnov test. Variables were presented as mean values and standard deviations if normally distributed, and otherwise, as median values (interquartile range). Categorical data were presented as number (frequency). The Student *t* test was used to determine the significance of variable differences between two time-points perioperatively. A Pearson or Spearman correlation test was performed to determine the correlation between plasma HNPs 1–3 data and clinical parameters (PaO_2_/FiO_2_ ratio and MV time). A stepwise multiple linear regression model was used to determine the independent risk factor for the clinical outcomes. The multivariate variables included age, weight, sex, CPB time, operation time, AC time and the plasma concentrations of HNPs 1–3, IL-6, IL-8 and IL-10. A *P* value <0.05 was considered statistically significant. All statistical analyses were performed by using SPSS (SPSS 16.0 for Windows; SPSS, Chicago, IL, USA).

## Results

### Study population

Thirty consecutive patients younger than 3 years who underwent cardiac surgery were finally enrolled from January 1 to 31, 2017, which included 18 (60%) infants. The general characteristics of the study patients are shown in Table 1. The cardiac defect of the patients included ventricular septal defect (VSD), VSD with atrial septal defect, VSD with patent ductus arteriosus, and tetralogy of Fallot. All patients underwent complete corrective surgery for cardiac lesion to ensure them no cyanosis after surgery. None of the cases need further surgical procedures for residual right to left shunt or other reasons. All of the children survived to hospital discharge.

**Table 1:** Demographic and clinical characteristics of the study population

Characteristic	
Sex male n (%)	17 (57%)
Age (months)	12.8 ± 8.9
Weight (kg)	7.9 ± 2.4
SpO2% before operation (%)	95 (88–99)
Pulmonary artery hypertension n (%)	11 (37%)
CPB time (minutes)	54.6 ± 17.6
Operation time (minutes)	106.4 ± 25.4
AC time (minutes)	33.6 ± 15.2
Ultrafiltration volume (ml)	33.7 ± 15.8
Mechanical ventilation time (hours)	12.6 ± 7.9
CICU LOS (days)	4.1 ± 1.5
Hospital LOS (days)	17.8 ± 5.8

Data are presented as number of patients (%), mean ± SD, or median (interquartile range), as appropriate

*SPO2* oxygen saturation, *CPB* cardiopulmonary bypass, *AC* aortic cross-clamp, *CICU* cardiac Intensive Care Unit, *LOS* length of stay

### Perioperative HNPs 1–3 and inflammatory cytokines concentrations in plasma

**Fig. 1:**
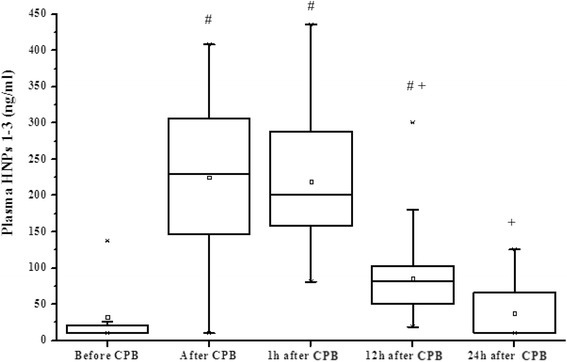
Perioperative courses over time of plasma HNPs 1–3 levels in the group. Data are presented as mean, median, 25th and 75th percentile range and minimum and maximal values. # Plasma HNPs 1–3 levels at different time points compares with that at the time before CPB; + Plasma HNPs 1–3 levels at different time points compares with that at the time after CPB. ^#, +^ *P* < 0.001. CPB, cardiopulmonary bypass

Plasma levels of HNPs 1–3 and the detected inflammatory cytokines are shown in Table 2. Plasma levels of HNPs 1–3 increased immediately after CPB (*P* < 0.001). While twenty-four hours after CPB, the levels of HNPs 1–3 decreased and approached almost to the baseline levels (Table 2 and Fig. 1).

**Table 2:** Perioperative plasma HNPs 1–3 and inflammatory cytokines levels

Variables	Before CPB	After CPB	1 h after CPB	12 h after CPB	24 h after CPB
HNPs 1–3 (ng/ml)	32.5 ± 44	224.9 ± 100.4^a^	220.4 ± 91.3^b^	85.6 ± 58.3^c,d^	37.7 ± 35.6^e^
IL-6 (pg/ml)	3.5 ± 6.3	17 ± 16.2^a^	68.3 ± 52.7^b,g^	139.7 ± 101^c,d^	136.2 ± 71.5^e,f^
IL-8 (pg/ml)	14.3 ± 6.1	37.8 ± 26.3	82.9 ± 70.5^h,k^	29 ± 17.5^i^	26 ± 14.6^j^
IL-10 (pg/ml)	24.2 ± 22.2	400.7 ± 236.5^a^	1525.7 ± 646.8^b,g^	66.4 ± 43.3^c,d^	32.1 ± 16.5^e^

Data are presented as mean ± SD

^a^ *P* < 0.001 after CPB versus before CPB;

^b^ *P* < 0.001 1 h after CPB versus before CPB;

^c^ *P* < 0.001 12 h after CPB versus before CPB;

^d^ *P* < 0.001 12 h after CPB versus after CPB;

^e^ *P* < 0.001 24 h after CPB versus after CPB;

^f^ *P* < 0.001 24 h after CPB versus before CPB;

^g^ *P* < 0.001 1 h after CPB versus after CPB;

^h^ *P* = 0.002 1 h after CPB versus before CPB;

^i^ *P* = 0.007 12 h after CPB versus before CPB;

^j^ *P* = 0.01 24 h after CPB versus before CPB;

^k^ *P* < 0.03 1 h after CPB versus after CPB

The plasma IL-6 and IL-10 levels increased immediately after CPB (*P* < 0.001), while IL-8 increased 1 h after CPB (*P* = 0.002). Twenty-four hours after CPB, the levels of IL-10 decreased near to the baseline levels, whereas IL-6 and IL-8 levels remained higher than the baseline levels (*P* ≤ 0.01). There were no significantly change in the plasma TNF-α and IL-1β levels during the selected perioperative times (data not show).

### Plasma HNPs 1–3 predict the severity and clinical outcome of lung injury

**Fig. 2:**
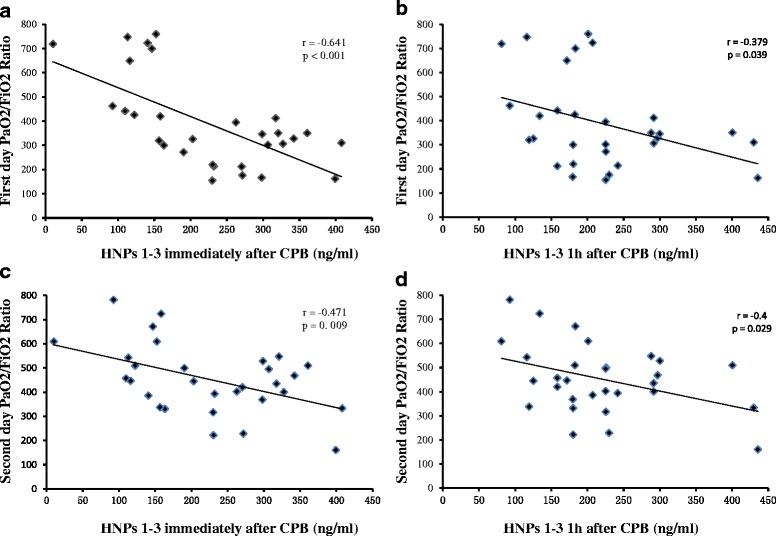
Scatterplot displaying the plasma HNPs 1–3 levels both at immediately after CPB and at 1 hour after CPB were significantly associated with more severe lung injury, as reflected by the PaO_2_/FiO_2_ ratio on the first 2 days after surgery. **a** and **b** for the first day: HNPs 1-3 (immediately after CPB): r = -0.641, P < 0.001; HNPs 1-3 (1 hour after CPB): r = -0.379, P = 0.039; **c** and **d** for the second day: HNPs 1-3 (immediately after CPB): r = -0.471, P = 0.009; HNPs 1-3(1 hour after CPB): r = -0.4, P = 0.029)

Higher levels of plasma HNPs 1–3 at two time points (immediately after CPB and at 1 h after CPB) were significantly associated with more severe lung injury, as reflected by the measurement of PaO_2_/FiO_2_ ratio on the first 2 days after operation (Fig. 2a-b for the first day: HNPs 1–3(immediately after CPB): *r* = −0.641, *P* < 0.001; HNPs 1–3(1 h after CPB): *r* = −0.379, *P* = 0.039; Fig. 2c-d for the second day: HNPs 1–3(immediately after CPB): *r* = −0.471, *P* = 0.009; HNPs 1–3(1 h after CPB): r = −0.4, *P* = 0.029). The plasma HNPs 1–3 levels significantly increased immediately after CPB, therefore the values at this time point were used for subsequent multivariable analysis. After adjusting for age, weight, sex, operation time, CPB time, AC time and the inflammatory cytokines (IL-6, IL-8 and IL-10) levels after CPB in a stepwise multiple linear regression model, we found that plasma HNPs 1–3 levels immediately after CPB was independently associated with the PaO_2_/FiO_2_ ratio on the first 2 days (for the first day: OR, −1.067, 95% CI, −0.548 to −1.574; P < 0.001; for the second day: OR, −0.667, 95% CI, −0.183 to −1.148; *P* = 0.009). [Additional file 3: Table S1 and Additional file 4: Table S2].

**Fig. 3:**
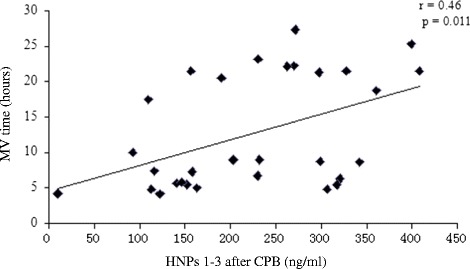
Scatterplot displaying the plasma HNPs 1–3 levels immediately after CPB enable prediction day the mechanical ventilation time after the surgery. CPB, cardiopulmonary bypass; MV, mechanical ventilation

Likewise, elevated plasma HNPs 1–3 levels immediately after CPB were correlated with longer MV time (Fig. 3, *r* = 0.46; *P* = 0.011). Even in the final multiple linear regression analysis, the HNPs 1–3 levels were still independent risk factors for longer MV time (OR, 0.039; 95% CI, 0.005 to 0.056; *P* = 0.011). [Additional file 5: Table S3].

## Discussion

In this prospective pilot study of the perioperative kinetics of plasma HNPs 1–3 levels in infants and young children undergoing cardiac surgery with CPB, we found that plasma concentrations of HNPs 1–3 remarkably increased immediately after CPB. The elevated levels were correlated with the severity of lung function decline (as reflect by PaO_2_/FiO_2_ on the first day, *r* = −0.641 and second day, *r* = −0.471 after surgery) and clinical outcomes of lung injury after cardiac surgery with CPB. Alarmin HNPs 1–3 could serve as an early predictor for lung injury after cardiac surgery.

HNPs are 29–33 amino acid cationic peptides containing six cysteines that form three canonical intermolecular disulfide bonds which stabilize the surface β-sheet motifs. HNP-1 and -3 differ only in the N-terminal amino acid, which, in HNP-1, is proteolyzed to generate HNP-2. There is no known functional difference among HNP-1 through −4 with respect to effects on host tissue [24, 29]. For HNP-1 through −3 comprise 97% of the total PMN pool, plasma HNPs 1–3 were measured as alarmin of onset of lung injury after cardiac surgery in our study. Previous studies provided supportive evidence that HNPs 1–3, increasing quickly in plasma and BALF [20, 22, 30], initiate the early events in the pathogenesis of acute lung injury in inflammatory lung disease [19, 22, 24, 25]. High levels of HNPs 1–3 suggested more severe lung injury [18, 20–22, 24]. As expected, in the current study, we found that plasma levels of HNPs 1–3 were significantly elevated immediately after CPB and the HNPs 1–3 levels were related with the impaired lung function, as reflected by the PaO_2_/FiO_2_ ratio on the first two days after cardiac surgery. After adjusting the clinical variants, which may impact this oxygenation index, HNPs 1–3 level remained as an independent risk factor for impaired oxygenation function after cardiac surgery. These findings suggested that plasma HNPs 1–3 levels could serve as a quantifiable alarmin for the post-CPB lung function decline in infants and young children. Unfortunately, to date, only a few studies have been conducted to HNPs 1–3 as an alarmin of lung injury, which were always based on small number of patients [20, 24]. Hence, a larger-cohort validation study is needed to verify these results in future. It should try to work out a cutoff value and/or predict positive value for lung dysfunction.

In addition, the present study found that plasma levels of HNPs 1–3 immediately after CPB were also positively correlated with the duration of MV (*r* = 0.46). Although speculative, the following reasons might explain these correlations. Recently, Liu et al. [26] reported that neutrophil-derived α-defensin had directly concentration-dependent pro-inflammatory and apoptotic effects in human bronchial and alveolar epithelial cells. It was verified by the latter fact that, in the transgenic mice, which express human a-defensin, neutrophil HNPs 1–3 contributes to the initiation of acute lung injury through low-density lipoprotein-related receptor-mediated disrupting capillary-epithelial barrier function within 4 h after acid aspiration [24]. The effects of HNPs 1–3 are not attenuated by plasma or tissue proteins that inhibit cytotoxicity, likely because of the high local concentrations attained when the protein is rapidly released from PMNs adherent to endothelial and epithelial cells [24, 29]. The facts indicate that HNPs 1–3 participate in the early events of lung injury directly. Intriguingly, in ARDS patients, the first twelve-hour plasma HNPs levels were found to closely correlate with the release of CRP, IL-8 and G-CSF in the circulation and with the severity of lung injury [20]. Otherwise, in vitro, stimulation of primary small airway epithelial cells with HNPs 1–3 induced expression of pro-inflammatory cytokines (IL-6, IL-8, IL-1β, IL-13, MCP-1 and 100A proteins), adhesion molecules (ICAM and VCAM), MMP-9 and β-defensins-2, but significantly decreased the expression of anti-inflammatory cytokine IL-10 [17, 22, 24, 26, 30, 31]. These cytokines were always reported to aggravate the HNPs 1–3 induced lung cell damage, lung (microvascular and epithelial) permeability and further diapedesis [1, 2, 4, 24, 28]. The infiltration of effector is mediated, at least in part, by the chemotactic activity of HNPs 1–3, the first characterized alarmin. The infiltrated cytotoxicity cells, attracted by HNPs 1–3, often perform synergistically effects with the multifunctional peptides on process pathophysiology of acute lung injury [22, 24, 25, 27]. On the other hand, importantly, HNPs 1–3 were reported to suppress neutrophil apoptosis via the P2Y_6_ signaling pathway resulting in the prolongation of their lifespan [27]. The prolonged survival of activated neutrophils in patients with ARDS, can cause the uncontrolled release of cytotoxic metabolites and pro-inflammatory substances (i.e., reactive oxygen species and proteases), which leads to the amplification of systemic inflammation, tissue injury and organ failure [27, 32]. Taken together, HNPs 1–3 contribute to more severe and prolonged inflammation in the lung and damage of the alveolar capillary barrier with increased lung water content and impaired oxygenation, inevitably resulting in longer MV support. There was no doubt that the HNPs 1–3 played an important role in the development of lung injury after cardiac surgery with CPB. It is necessary to prove that HNPs 1–3 are mediators of lung injury in patients after cardiac surgery and not merely makers of disease in future.

Notably, HNPs 1–3 gene polymorphisms have been found to affect the expression level and associate with lung function in pathologic states [21, 29, 33, 34], it should be emphasized that genetic variation of HNPs 1–3 must be considered in future. Further studies aimed at elucidating the aforementioned issues would help gain additional insight into the mechanism by which these alarmins modulate lung injury after cardiac surgery.

Previous studies have shown that normal plasma contained miniscule amounts of HNPs 1–3, usually below 100 ng/ml, and although the concentration of HNPs 1–3 was considerably higher in plasma of patients with sepsis or inflammatory lung disease, most of these peptides would be protein bound [24, 29, 35]. In the current pilot study, we found similar kinetics of plasma HNPs 1–3 perioperatively in children that the elevated plasma HNPs 1–3 levels descend twenty-four hours after operation. The notoriously ‘non-specific’ binding proclivities may, to some extent, account for the short lifetime of HNPs 1–3 in plasma. No degradative pathway for their removal from tissue has been identified. Another possible explanation for this decrement is the ingestion of HNPs 1–3 by peripheral blood mononuclear cells, for previous studies have shown that these cells can capture defensins in various ways after a much mount of HNPs suddenly released into the circulation [29, 36]. In addition, the expression of α-defensins significantly correlated with the proportion and number of airway neutrophils [19]. Although patient with relative higher levels HNPs 1–3 concentration in patients had longer MV time in the study, factors activating neutrophil gradually disappeared with the patients weaning from the ventilator, which down regulated the release HNPs 1–3 from neutrophil [3, 9]. Interestingly, plasma HNPs 1–3 concentrations returned to the normal range after pulmonary inflammatory disease was treatment, which followed with reduction of the activated neutrophil count [37].

Several issues of the present study should be noticed with caution. First, we used the current American-European Consensus Conference (AECC) definitions for acute lung injury and ARDS to definite the impaired lung function regardless of FIO_2_ or PEEP. Although the predict value of the alarmin HNPs 1–3 for the occurrence of acute lung injury in this study is consistent with that in previous published studies [17, 18, 20, 22], our relative weak correlation findings were based on a small number of patients in a single center, which might limit the application of these findings to other institutions. Furthermore, we enrolled the patients underwent surgery for CHD in the absence of relevant comorbidities and particularly of known lung disease. Therefore, we are not sure whether the HNPs 1–3 changes would be the same in the presence of previous lung diseases. Because children often have pneumonias before cardiac operations, future studies are needed to confirm this. Third, the HNPs 1–3 levels in BALF were not measured in this study. However, the intriguing findings indicated that elevated of HNPs 1–3 in plasma significantly correlated with the proportion and number of airway neutrophils [17, 19, 37]. In addition, the accumulation of airway neutrophils is reported to be directly associated with the activation state of circulating neutrophils [19, 20], which, to some extent, suggests that HNPs 1–3 in pulmonary may have the similar kinetics and functions in lung injury.

## Conclusions

We found that plasma HNPs 1–3 could act as a quantifiable alarmin biomarker for onset of lung injury after cardiac surgery with CPB in infants and young children. The plasma levels of HNPs 1–3 are associated with lung function and clinical outcomes after CPB. Insight into the role of the HNPs 1–3 in the post-cardiac surgery inflammatory response holds the potential for better understanding of pulmonary dysfunction induced by cardiac surgery necessitating CPB.

## Additional files

Additional file 1: Standard anesthesia and cardiopulmonary bypass protocol. (DOCX 14 kb)

Additional file 2: Ventilator settings and Wean from mechanical ventilation protocol. (DOCX 14 kb)

Additional file 3: Table S1. Multiple linear regression model analysis independent risk factors associated with PaO2/FiO2 ratio on the first day after CPB operation. (DOCX 15 kb)

Additional file 4: Table S2. Multiple linear regression model analysis independent risk factors associated with PaO2/FiO2 ratio on the second day after CPB operation. (DOCX 15 kb)

Additional file 5: Table S3. Multiple linear regression model analysis independent risk factors associated with prolonged MV time after CPB operation. (DOCX 15 kb)

## Abbreviations

AC: Aortic cross-clamp
ARDS: Adult respiratory distress syndrome
BALF: Bronchoalveolar lavage fluid
CHD: Congenital heart disease
CICU: Cardiac Intensive Care Unit
CPB: Cardiopulmonary bypass
CPE: Cardiogenic pulmonary edema
EF: Ejection fraction
HNPs 1–3: Human neutrophil peptides (HNPs) 1–3
LOS: Length of stay
MV: Mechanical ventilation
PMNs: Polymorphonuclear leukocytes
RACHS-1: The Risk Adjusted Classification for Congenital Heart Surgery
SpO_2_: Pulse oximetry saturation
