# Supplementary material for: Plasma levels of alarmin HNPs 1–3 associate with lung dysfunction after cardiac surgery in children
Source: BMC Pulm Med. 2017 Dec 28;17:218. doi: 10.1186/s12890-017-0558-4 (PMC5745992; doi:10.1186/s12890-017-0558-4)
Supplement: Supplementary file 1 — Standard anesthesia and cardiopulmonary bypass protocol. (DOCX 14 kb) [file 12890_2017_558_MOESM1_ESM.docx]

**Standard anesthesia and cardiopulmonary bypass protocol**

All patients were evaluated by standard echocardiography or/and cardiovascular angiography before surgery. The patients were orally intubated in the operating room. Anesthesia was managed according to a standard protocol, including induction with ketamine (2mg/kg) and midazolam (0.2mg/kg) and maintenance with fentanyl (25-45μg/kg) and isoflurane in oxygen. Neuromuscular blockade was achieved with vecuronium (0.1mg/kg, once every 40 min).

The CPB circuit, which was identical for all patients, included a microporous hollow fiber membrane oxygenator (Dideco 901, Dideco Liliput, Italy; Medtronic, Inc, Minneapolis, USA) and a Stockert III roll pump (Stockert Instrumente; Munich, Germany). Before aortic cannulation, 400-450 U/kg heparin was administered with the target kaolin-ACT value more than 450s. The bypass circuit was primed with lactated Ringer's solution, colloid (20% albumin, plasma 150 mL), mannitol (2.5 mL/kg), packed red blood cells (1.5U), heparin (1000 IU for Dideco 901; 1250 IU for Medtronic), and 5% sodium bicarbonate (5 mL/kg). Pump flow rates ranged from 3.0 to 2.0 L·min^-1^· m^-2^. Core temperature was controlled at 30-32°C using a heat exchanger in the bypass circuit. At the end of CPB, in order to maintain the fluid balance, the modified ultrafiltration was used to remove the excess fluid in the body according to the hematocrit (maintenance of hematocrit >30%) and the monitored blood pressure (Aortic blood pressure: 75-110/50-78 mmHg; Left atrial pressure: 5-12 mmHg; Right atrial pressure: 5-14 mmHg according to the patient’ age and weight).
