# Supplementary material for: Plasma levels of alarmin HNPs 1–3 associate with lung dysfunction after cardiac surgery in children
Source: BMC Pulm Med. 2017 Dec 28;17:218. doi: 10.1186/s12890-017-0558-4 (PMC5745992; doi:10.1186/s12890-017-0558-4)
Supplement: Supplementary file 2 — Ventilator settings and Wean from mechanical ventilation protocol. (DOCX 14 kb) [file 12890_2017_558_MOESM2_ESM.docx]

**Ventilator settings and Wean from mechanical ventilation protocol**

The patients were transferred to the surgical ICU immediately after operation and mechanical ventilated using Servo i ventilators (Siemens; Munich, Germany). In our center, the settings of PEEP and FIO_2_ were up to the discretion of the managing clinician according the clinical practice requirements. The initial mode of ventilation was pressure-regulated volume control with the ratio of the duration of inspiration to the duration of expiration (I/E) of 1:2 in all patients. The inspiratory oxygen fraction (FIO2) was 30%-100% and was thereafter reduced to the lowest value which maintains pulse oximetry saturation (SpO2) higher than 95%. When FIO2 was higher than 60%, PEEP was increased 2cmH2O every 2 hours with/ without dilating the lung until the PEEP reached to 9 cmH2O. When FIO2 was lower than 60%, PEEP was reduced 1cmH2O every 1 hour until the PEEP lower than 5 cmH2O. Tidal volume was 6-8 ml/kg maintain peak inspiratory pressure lower than 30 cmH2O. The respiratory frequency was 22-30 breath/min. Once the patient was breathing spontaneously and ready for weaning, the ventilator mode was switched to pressure-controlled, synchronized, intermittent, mandatory ventilation. The tidal volume reduced to 4-6 ml/kg. All these parameters were adjusted to maintain a carbon dioxide tension of 35 mmHg.

Patients were weaned from mechanical ventilation (MV) when they met the following criteria: stable hemodynamic profile, normal cardiac rhythm, adequate oxygenation on fraction of inspired oxygen≤0.4, maintenance of pH>7.35 and PaCO_2_<45 mmHg, the level of consciousness consistent with adequate airway protective reflexes, absence of accessory respiratory muscle recruitment, and approval by the attending cardiac intensivists.
