# Supplementary material for: Plasma levels of alarmin HNPs 1–3 associate with lung dysfunction after cardiac surgery in children
Source: BMC Pulm Med. 2017 Dec 28;17:218. doi: 10.1186/s12890-017-0558-4 (PMC5745992; doi:10.1186/s12890-017-0558-4)
Supplement: Supplementary file 3 — Multiple linear regression model analysis independent risk factors associated with PaO2/FiO2 ratio on the first day after CPB operation. (DOCX 15 kb) [file 12890_2017_558_MOESM3_ESM.docx]

**Additional File 3: Table S1.** Multiple linear regression model analysis independent risk factors associated with PaO2/FiO2 ratio on the first day after CPB operation.

| Variables | Odd Ratio | 95% Confidence  Interval | P Value |
| --- | --- | --- | --- |
| T2 | -1.067 | -0.548— -1.574 | < 0.001 |
| CPB | -3.797 | -0.865 — -6.728 | 0.013 |
