# Supplementary material for: Plasma levels of alarmin HNPs 1–3 associate with lung dysfunction after cardiac surgery in children
Source: BMC Pulm Med. 2017 Dec 28;17:218. doi: 10.1186/s12890-017-0558-4 (PMC5745992; doi:10.1186/s12890-017-0558-4)
Supplement: Supplementary file 4 — Multiple linear regression model analysis independent risk factors associated with PaO2/FiO2 ratio on the second day after CPB operation. (DOCX 15 kb) [file 12890_2017_558_MOESM4_ESM.docx]

**Additional File 4: Table S2.** Multiple linear regression model analysis independent risk factors associated with PaO2/FiO2 ratio on the second day after CPB operation.

| Variables | Odd Ratio | 95% Confidence  Interval | P Value |
| --- | --- | --- | --- |
| T2 | -0.667 | -0.183— -1.148 | 0.009 |
