# Supplementary material for: Plasma levels of alarmin HNPs 1–3 associate with lung dysfunction after cardiac surgery in children
Source: BMC Pulm Med. 2017 Dec 28;17:218. doi: 10.1186/s12890-017-0558-4 (PMC5745992; doi:10.1186/s12890-017-0558-4)
Supplement: Supplementary file 5 — Multiple linear regression model analysis independent risk factors associated with prolonged MV time after CPB operation. (DOCX 15 kb) [file 12890_2017_558_MOESM5_ESM.docx]

**Additional File 5: Table S3.** Multiple linear regression model analysis independent risk factors associated with prolonged MV time after CPB operation.

| Variables | Odd Ratio | 95% Confidence  Interval | P Value |
| --- | --- | --- | --- |
| T2 | -0.039 | -0.005— -0.056 | 0.011 |
| CPB | -0.174 | -0.028 — -0.032 | 0.021 |
